# Process Modelling and Simulation of Key Volatile Compounds of Maillard Reaction Products Derived from Beef Tallow Residue Hydrolysate Based on Proxy Models

**DOI:** 10.3390/foods11192962

**Published:** 2022-09-22

**Authors:** Jingwei Cui, Yinhan Wang, Huihuang Zhang, Jiulin Li, Qiaojun Wang, Lixue Yang, Hui Zhang, Qingzhe Jin, Gangcheng Wu, Xingguo Wang

**Affiliations:** 1 State Key Laboratory of Food Science and Technology, School of Food Science and Technology, National Engineering Research Center for Functional Food, International Joint Research Laboratory for Lipid Nutrition and Safety, Collaborative Innovation Center of Food Safety and Quality Control in Jiangsu Province, Jiangnan University, Wuxi 214000, China; 2 School of Aerospace Engineering, Beijing Institute of Technology, Beijing 100081, China; 3 International School, Beijing University of Posts and Telecommunications, Beijing 100876, China; 4 Guanghanshi Maidele Food Co., Ltd., Guanghan 618300, China

**Keywords:** hydrolysis time, Maillard reaction products, cubic spline interpolation, polynomial curve fitting, curve prediction model

## Abstract

The hydrolysis time is directly related to the flavor of the Maillard reaction, but existing proxy models cannot simulate and model the variation curves of vital volatile components. This study developed a predictive model for modelling and simulating key volatile compounds of Maillard reaction products (MRPs) derived from beef tallow residue hydrolysate. Results showed the degree of hydrolysis increased with hydrolysis time, and the most significant improvement in the roast flavor and overall acceptance was when hydrolyzing 4 h. Based on flavor dilution value and the relative odor activity value, nine key volatile components were identified, and 2-ethyl-3,5-dimethylpyrazine with roast flavor was the highest. Compared with Polynomial Curve Fitting (PCF) and Cubic Spline Interpolation (CSI), key volatile compounds of MRPs could be better modeled and simulated by the Curve Prediction Model (CPM). All results suggested that CPM could predict the changes in key volatile components produced by MRPs.

## 1. Introduction

Beef tallow is mainly processed from suet, a thick fat film on the ribs of cattle abdomen and dislodged after slaughtering cattle. As a result of its unique flavor, beef tallow is preferred for baking and serving as a hotpot bottom material by the catering industry. After frying and squeezing suet, beef tallow residue contains a significant proportion of protein. Most of the beef tallow residues serve no purpose. Some are fed to chickens, while others are buried, resulting in a waste of resources and environmental pollution.

The use of meat flavors has been widely conducted in the food industry, including general flavor and characteristic flavor. Plant, yeast, or animal proteins can generate meaty flavor in Maillard reactions [1]. Nevertheless, the meat flavor produced by plant proteins and yeast protein by Maillard reaction shows weak acridity. In contrast, through enzyme hydrolysis, products of the Maillard reaction with animal proteins possess pure and rich meat aromas when appropriate conditions are met [2]. Compared with other factors, including protease dosage, pH and solid-to-liquid ratio, enzymatic hydrolysis could influence the flavor of Maillard reaction products. Weng et al. found that the content of 1-octene-3-ol (soybean odorous substance) was effectively reduced by the optimal enzymatic hydrolysis, while the deterioration of enzymatic hydrolysates flavor occurred from the excessive enzymatic hydrolysis [3]. Wang et al. also reported that the content of volatile compounds, including esters, acids, alcohols and aldehydes, was significantly improved by the hydrolysis of pork sarcoplasmic protein and myofibrillar protein [4]. However, the effect of hydrolysis on the volatile compounds of Maillard reaction products derived from beef tallow residue is still unknown.

However, several factors can affect the process. For instance, some scholars found that hydrolysis was affected by many factors: hydrolysis time, protease dosage, hydrolysis temperature, pH, solid-to-liquid ratio, etc., that affect the hydrolysis of Litopenaeus vannamei [5]. This study concluded that the essential factor was hydrolysis time. According to the research of some scholars, soybean protein immunoreactivity increased and decreased with different hydrolysis times [6]. They found that controlled hydrolysis conditions combined with controlled Maillard induction did not significantly decrease the immune activity.

Partial least squares regression (PLSR) has been commonly used to predict the Maillard reactions with different degrees of hydrolysis (DH) based on a point-to-line basis. Some studies carried out a correlation analysis between the molecular weight of peptides, odor-active compounds and sensory evaluation of beef with various DH by using PLSR and found that beef matrix with DH 29.13 was an ideal precursor to impart aroma characteristics of beef processing flavor [7]. Some researchers developed a PLSR model based on Fourier transform infrared spectroscopy to predict DH using the molecular weight of milk protein hydrolysate, indicating that PLSR is a promising tool for the prediction of DH in the Maillard reactions [8]. Although PLSR is precise and sensitive, the large amount of experimental data required makes it time-consuming. Recently, proxy models have become popular with the development of computation, including Cubic Spline Interpolation (CSI), Polynomial Curve Fitting (PCF) and Curve Prediction Model (CPM). Proxy models could resolve the sequence integrity and low-reliability issues without requiring a large sample space of data and also generate irregular raw data to construct an intense generated sequence to make it highly accurate and sensitive in dealing with a small amount of data. For example, CSI could handle curve simulation with sampled points and has ideal geometric characteristics, smooth curves, high fitting precision and practicability [9], and the effect of temperature on crop yields could be well predicted by CSI [10]. For PCF, it provides discontinuity and asymmetry in fitting curves, which are suitable for anatomical curves [11]. Although the PCF, CSI and CPM had been applied in food-related fields such as irrigated food planting, the key volatile compounds in the Maillard reaction modelling and simulating with PCF, CSI and CPM have not been studied.

Based on the above discussion, the purpose of this study was to perform modelling and simulations of key volatile compounds responsible for the Maillard reaction in beef tallow residue hydrolysates using different proxy models. Gas chromatography-olfactometry-mass spectrometry analysis (GC-O-MS) and aroma extract dilution analysis (AEDA) were qualitative and quantitative analyses of the key volatile components. It was also performed by ROAV for sensory evaluation. A relationship between the volatile components at the Maillard reaction products (MRPs) of beef tallow residue and time was investigated using the CPM, CSI and PCF.

## 2. Materials and Methods

### 2.1. Chemicals and Materials

Beef tallow residue (69% protein, 17% lipid, 3% water) was obtained from Guanghanshi Maidele Food Co., Ltd. (Deyang, China). Tanggui China Co., Ltd. delivered Flavourzyme (50,000 U/mg). Other chemical reagents were purchased from National Chemical Reagent Co., Ltd. (Shanghai, China).

### 2.2. Preparation of Beef Tallow Residue Hydrolysates

A total of 5 g of beef tallow residue was ground with an FW80 grinder (Scientific Instrument Co., Ltd., Shanghai, China) into powder, and then 0.15 g of flavourzyme was added, mixing with deionized water in 1:4 ratios with stirring at room temperature. Then its hydrolysis was performed with flavourzyme for different time intervals of 0 h, 2 h, 4 h, 6 h and 8 h. All five hydrolysates were prepared under optimal hydrolysis conditions (40 °C). Inactivation of protease in each of the five hydrolysates (A, B, C, D and E) was performed by heating them to 100 °C for 15 min, then centrifuging them with DT5-1 centrifuge (Scientific Instrument Co., Ltd., Shanghai, China) at 3800× *g* for 20 min. The supernatant was separated, and around 2 g of the supernatant was freeze-dried at −80 °C for 24 h before storing at 4 °C for further experiments. All the experiments were repeated three times.

### 2.3. Measurement of DH

DH is determined by dividing the number of broken peptide bonds by the total number of bonds to express the degree of free amino acids during hydrolysis. A formaldehyde titration was used to determine the amino nitrogen content [12]. We added 20 mL of distilled water to the hydrolyzed tallow residue supernatant and adjusted the pH to 7.0 with 0.1 mol/L NaOH. Afterward, 10 mL of 38% (*v*/*v*) formaldehyde solution was added to the beaker, and the pH was adjusted to 9.5 with 0.1 mol/L standard NaOH solution. Determination of total nitrogen content was by the Kjeldahl method (AOAC 991.20). The value of DH was calculated according to the following equation:

$$\mathrm{DH}=\frac{C\times \left(V_{1}-V_{2}\right)\times V/5}{m\times p\times 8}\times 100$$

where *C* is the concentration of standard titration NaOH. *V*_1_ is the consumed volume of 0.05 M NaOH with titrating from pH 8.2 to pH 9.2 for beef tallow residue hydrolysate, while *V*_2_ is the consumed volume of 0.05 M NaOH with titrating from pH 8.2 to pH 9.2 for distilled water. *V* is the total volume of beef tallow residue hydrolysate, and *m* is the mass of the raw material. For *p*, it is the percentage of protein in raw material [13]. All analyses were repeated three times to assess the results.

### 2.4. Preparation of MRPs

For the Maillard reaction, reaction time (20–80 min), Xylose addition (2.0–5.0%) and reaction temperature (60–120 °C) have been optimized in our pre-experiment according to the sensorial results obtained. The optimized Maillard reaction condition was reaction time 60 min, Xylose addition 3% and reaction temperature 100 °C, and the MRPs were prepared under the optimal Maillard reaction conditions. The MRPs (marked A, B, C, D, E) were obtained by mixing 10 mL of the hydrolysates and 0.30 g xylose in a beaker, followed by heating in an oil bath at 100 °C for 1 h. These five MRPs were cooled to room temperature using cold water after the Maillard reaction and then stored in a refrigerator at 4 °C for further use.

### 2.5. Aroma Extract Dilution Analysis by GC-O-MS

A GC–MS (QP-2010; Shimadzu, Kyoto, Japan) combined with an olfactory port was employed to analyze the samples. GC–O analysis was performed on a DB-WAX column (30 m × 0.25 mm × 0.25 μm); and experimental conditions were in accordance with GC–MS. Headspace solid phase microextraction (HS-SPME) extracted the volatile from samples, HS-SPME was measured according to 2.6. The samples were diluted by increasing the GC inlet split ratio from 3:1 to 9:1, 27:1 and 81:1, respectively. Aroma extracts were orthonasally evaluated by three trained sensory panelists to describe the odor, and each panelist repeated it twice. An experienced laboratory technician recorded the retention time and odor descriptions of the sensory panelists. The flavor dilution (FD) factor was defined as the maximum dilution where the aroma compound could be detected.

### 2.6. Relative Quantification of Aromatic Compounds

The HS-SPME method was based on the previous research with some modifications [14]. The MRPs (2.0 ± 0.02 g) were placed in 20-mL flasks sealed using a metallic cap, and a 50 μm/30 μm DVB/CAR/PDMS fiber (Supelco Inc., Bellefonte, PA, USA) was used for headspace sampling. Volatile compounds were analyzed by GC–MS using a TSQ Quantum XLS (Thermo, Waltham, MA, USA) operating in electron ionization mode (EI, 70 eV). Helium (He, purity > 99.999%) was used as the carrier gas, flowing at 1.8 mL/min. HS-SPME analysis was performed under splitless injection, and the mass spectrum was detected in the selected ion monitoring (SIM) mode. An automatic deconvolution and identification system (AMDIS) was used to analyze the n-alkane C7–C40 data file to obtain the n-alkane retention index. The peaks were obtained from GC-MS analysis, and the total ion current of the peaks was searched through Wiley 6.0 (Wiley, New York, NY, USA) and NIST 98 (National Institute of Standards and Technology, Gaithersburg, MD, USA). The relative content of volatile compounds can be estimated by comparing the peak area of the volatile compounds to the total peak area. Details of GC-MS conditions from other studies were referenced [14].

### 2.7. Main Volatile Flavor Compounds Based on ROAV Analysis

Based on the relative quantification of each volatile component and according to the fragrance threshold of each volatile substance in water in references, we calculated the ROAV of each component according to the following formula:

$${\mathrm{ROAV}}_{i}=\frac{C_{i}}{C_{max}}\times \frac{T_{max}}{T_{i}}\times 100$$

where *C_i_* was the relative content (%) of volatile component *i*, and *T_i_* was the fragrance threshold (µg/kg) of component i in water. *C_max_* and *T_max_* were the relative content (%) and fragrance threshold (µg/kg) of the component that contributed the most to the total flavor of the samples (2-ethyl-3,5-dimethylpyrazine).

The fractions with roav ≥ 1 were considered the main flavor compounds in MRPs, and the fractions with 0.1 ≤ roav ≤ 1 were considered to have an essential modification to the overall flavor of MRPs.

### 2.8. Sensory Evaluation

A total of 12 trained panelists (6 males and 6 females, aged 20–28) were recruited from Jiangnan University. The MRPs were placed in paper cups marked with 3-digit random numbers and were presented to each assessor. Panelists were trained by identifying and describing the odor quality of standard odorants. All the panelists received five extensive trainings with reference to other studies in the training procedure [15]. After the training, the panelists could successfully identify and describe the relevant odor. They all had conducting sensory evaluation experience and were experienced with quantitative description evaluation methods. The sensory evaluation of beef tallow residue MRPs was conducted on five samples. According to the method of Xu et al. [16,17], several flavor characteristics were selected using descriptive testing before the analysis. It results in providing a reference solution in water for each characteristic sensory descriptor: green (hexanal, 3 ppm), rancid (hexanoic acid, 3 ppm), fatty ((E)-2-nonenal, 3 ppm), fruity (β-cyclocitral, 3 ppm), stale (benzene acetaldehyde, 3 ppm) and roasty (2,6-dimethylpyrazine, 3 ppm). Trained sensory specialists rated each item on a linear scale from 0 (non-perceivable) to 10 (strongly perceivable). Consumers rated the overall acceptability of the samples based on appearance, flavor, taste and texture. A five-point hedonic scale (5 = like very much, 4 = like moderately, 3 = neither like nor dislike, 2 = dislike moderately and 1 = dislike very much) was used. When the result of sensory evaluation was more than 3 points, these samples were considered as the overall acceptance. Each panelist evaluated each sample three times.

### 2.9. Mathematical Modelling

Volatile components showed a complex nonlinear relationship with time. Three proxy models were used to simulate the relation curve. Based on experimental results, the proxy model was adjusted or trained.

(1) CPM: Using initial values and 100 arithmetic progressions within the interval of independent variables, the predictive model fitted the curve. The independent variable predicted each dependent variable and was used for curve fitting.
(2) CSI: To achieve the curve drawing, we used many function constraints, including:
  An internal node on the curve should have equal left and right values.
  The function’s first and last end points should appear in the corresponding equation.
  Each end of the node must have the exact derivative.
  Both ends of the node should have the same second derivative.
  The second derivative at the end point should be zero [18].
(3) PCF: Taking the partial derivative of the introduced coefficient and then making the partial derivative 0 allowed us to fit the curve by minimizing the square of the residuals between the dependent variables [19].

### 2.10. Verification Experiment

We calculated the ROAVs using Methods Section 2.5 and Section 2.6 based on the relative content of the volatile components over the fifth hour. We then calculated the error for each proxy model using the following formula

$$Error=ABS\left(R_{a}-R_{p}\right)$$

where *R_a_* represents the actual ROAVs, and *R_p_* indicates the predicted ROAVs.

### 2.11. Statistical Analysis

Data from the DH, volatile components and sensory evaluation were analyzed using SPSS version 13.0 (SPSS Inc., Chicago, IL, USA). Python 3.7 analysis software was used, with drawing by matplotlib-3.5.1. The model was built based on scipy-1.8.0, MATLAB 2020, PyCharm 2021.3.3-Windows and TensorFlow-1.13.0. Significant differences among groups were performed statistically by one-way analysis of variance (ANOVA) combined with Tukey’s multiple-range test using SPSS (*p* ≤ 0.05).

## 3. Results

### 3.1. Analysis of DH in Hydrolysate

We hydrolyzed beef tallow residue for 0 h, 2 h, 4 h, 6 h and 8 h, respectively (DH 0.00 ± 0.00, 9.40 ± 0.24, 11.93 ± 0.13, 14.32 ± 0.21 and 14.77 ± 0.46). The DH of the hydrolysate of beef tallow residue was significantly different among different groups (*p* ≤ 0.05). A significant increase in DH was observed with hydrolysis time. Meanwhile, the DH growth rate slowed down with the extension of hydrolysis time.

### 3.2. Overall Aroma Evaluation

As shown in Table 1, 30 aroma-active compounds, including 13 aldehydes, 6 acids, 3 alcohols, and 3 pyrazines, were identified in MRPs with GC-MS, and the flavor of these compounds was mainly manifested as fatty, fruity, green, roasty, stale and rancid. Compared with other volatile compounds, more aldehydes were produced; (E)-2-octenal and octanal resulted in fatty in MRPs, while (E,E)-2,4-decadienal, (E,E)-3,5-octadien-2-one, 1-octen-3-ol and benzene acetaldehyde caused MRPs to produce an unpleasant taste. The acids of MRPs such as n-decanoic acid, butanoic acid, octanoic acid, heptanoic acid, hexanoic acid and nonanoic acid showed rancid flavor. With the prolongation of hydrolysis time, the FD values of many flavors active compounds, such as (E,E)-2,4-nonadienal and ethyl myristate decreased, while the FD values of many flavors active compounds, such as octanal, decanal, 2-Ethyl-3,5-dimethylpyrazine and heptanal increased; 2-ethyl-3,5-dimethylpyrazine exhibited the highest FD value (≥81) in MRPs. In addition, the other key volatile flavor components, such as (E)-2-Nonenal and benzene acetaldehyde, also had higher FD values (27) in MRPs.

### 3.3. Identification of Aroma-Active Compounds

Table 2 and Table 3 list the relative content and ROAVs for volatile compounds in MRPs, respectively. ROVAs of 10 aroma-active compounds in MRPs were higher than one, and 2-ethyl-3,5-dimethylpyrazine had the highest ROAVs of all five samples, which was consistent with AEDA results. ROVAs of the acids were lower than 0.1, while those of aldehydes were higher than 0.1. Generally, the relative content of aldehydes, alcohols and furans was high, while that of pyrazines and acids was low. The formation of some active flavor compounds was related to DH, such as butyric acid, trimethyl-pyrazine and p-cresol, which was generated with the increase of DH.

### 3.4. MRPs Sensory Analysis

**Figure 1:**
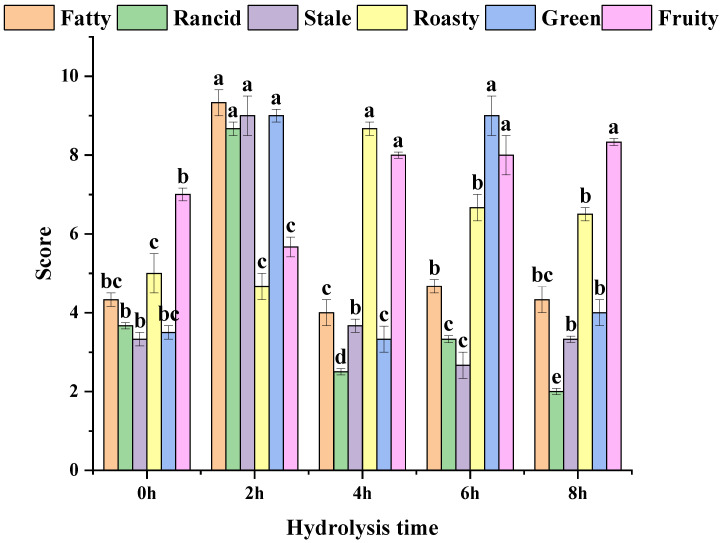
The result of sensory evaluation among MRPs. The values followed by different letters were significantly different (*p* ≤ 0.05).

**Figure 2:**
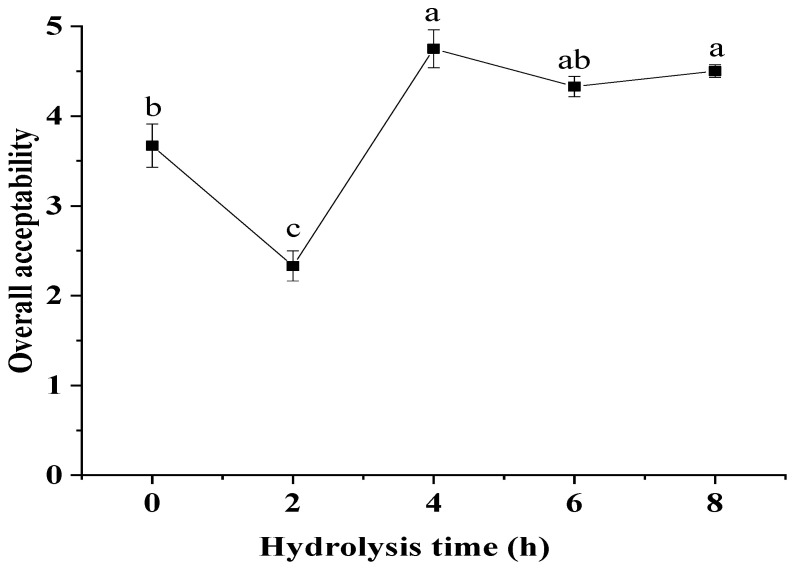
The overall acceptability of MRPs. The values followed by different letters were significantly different (*p* ≤ 0.05).

The flavor of MRPs was related to the key volatile components, which indicated that it could influence the quality of MRPs over hydrolysis time. As shown in Figure 1 and Figure 2, each flavor and overall acceptability changed significantly with the prolongation of hydrolysis time. The flavor was the best in MRPs C hydrolysis because it had the highest roast flavor and the least stale flavor. The highest rancid, fatty, stale and lowest roasty were observed MRPs B, which resulted in the worst flavor performance. MRPs D had the lowest stale, while it had the highest green. MRPs E showed the highest fruity and the lowest rancid, but it had a less roasty flavor. When it was not hydrolyzed, each flavor score was low. The fatty, rancid, stale and green increased with the increase of DH. After hydrolyzing for 4 h, the overall acceptability was increased. With the prolongation of hydrolysis time, the roast flavor which had the greatest effect on the overall acceptability first increased and then decreased.

### 3.5. Simulation of Key Volatile Components Curve

**Figure 3:**
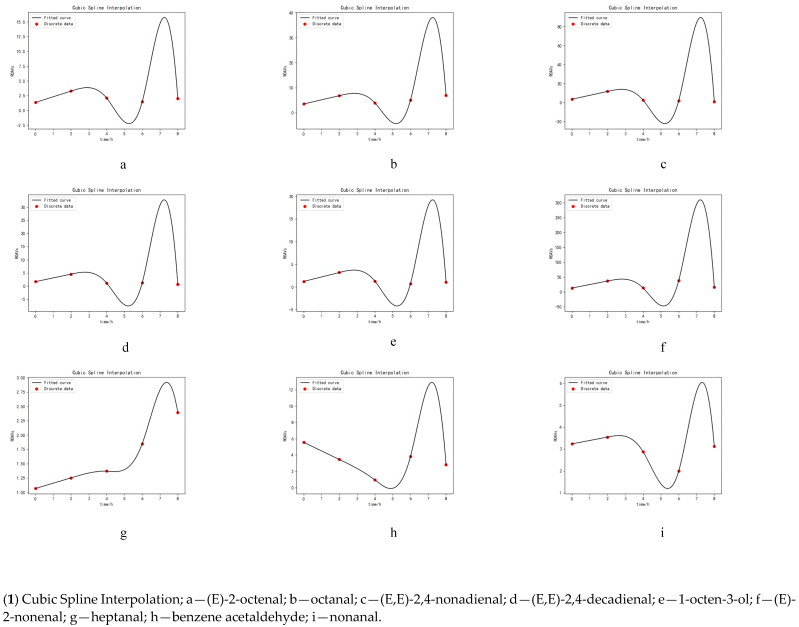

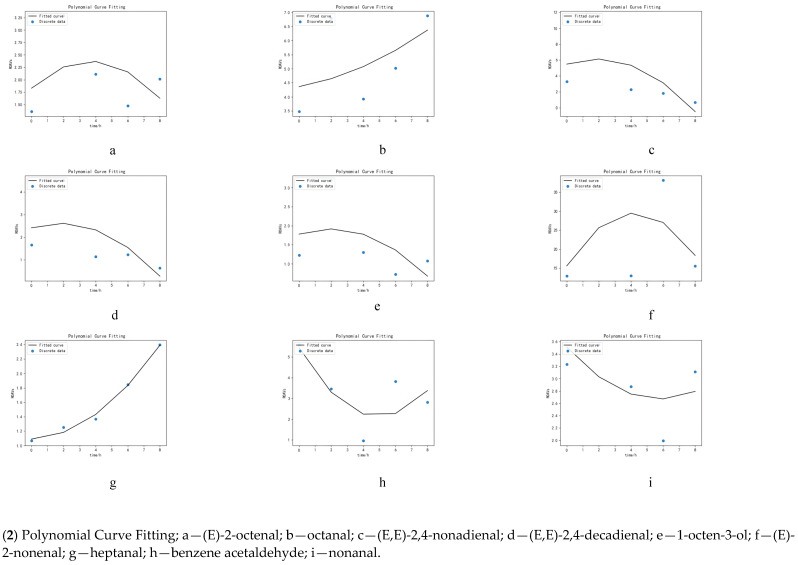

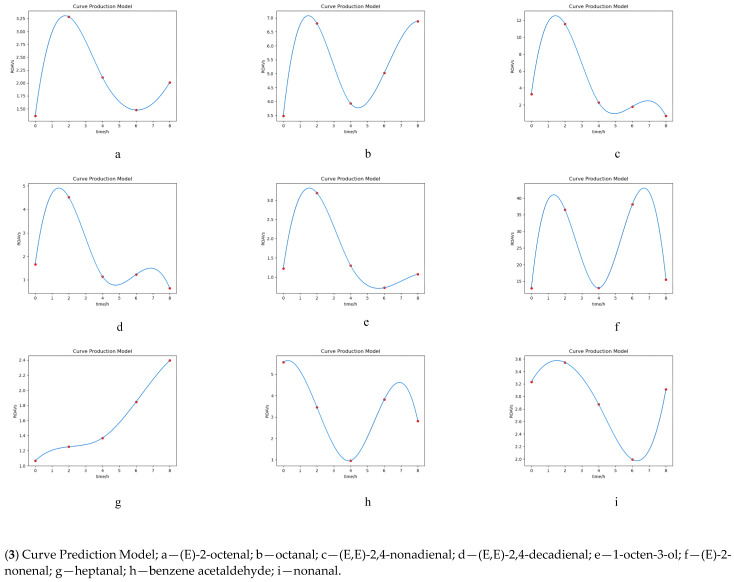
Fitting curves by different proxy models. Simulation of volatile component curves by three models: (**1**) Cubic Spline Interpolation; (**2**) Polynomial Curve Fitting; (**3**) Curve Prediction Model.

Nine key volatile components (ROAVs > 1) that contributed significantly to the flavor were selected for curve simulation. As shown in Figure 3, different methods had different fitting effects on the time-volatile component curve. The PCF was not smooth, similar to the combination of many straight segments. The PCF simulation curve results showed that each point fell on the curve only in group heptanal. In contrast, the points of other groups were distributed on both sides of the curve. In addition, the (E,E)-2,4-nonadienal of PCF reported negative values at high DH. The (E)-2-nonanal, (E,E)-2,4-decadienal, (E,E)-2,4-nonadienal, 1-octene-3-ol and octanal groups of CSI reported negative values with the increase in DH. In CSI, there was strong oscillation at both ends of the interpolation, showing either a sharp increase or decrease between the two measured points, which leads to a large fluctuation of the function. In contrast, the CPM was smooth with no negative values.

### 3.6. Validation Experiment of Volatile Components Curve

As shown in Table 4, each curve’s predictions of the volatile components in the fifth hour were different, with the CPM having the highest accuracy, PCF having the second-highest accuracy and CSI having the lowest accuracy. PCF had the best prediction effect on heptanal (0.07) and the worst prediction effect on (E,E)-2,4-nonadienal (3.233). CSI was the best predictor of octanal (0.001), with negative predictions for (E,E)-2,4-nonadienal (−21.173), (E,E)-2,4-decadienal (−6.91), 1-octen-3-ol (−3.742) and (E)-2-nonenal (−45.821). The overall error of CPM was small, and the curve fitting was accurate. By comparison, it could be seen that CPM was more suitable for modelling and simulation of the key volatile compounds in the Maillard reaction of beef tallow residue hydrolysate.

## 4. Discussion

In flavor science, the combination of GC-O-MS and AEDA has proven a valuable tool for identifying and ranking key odors in various foods. The aldehydes were most abundant among the 30 volatile components identified by GC-O-MS after the fat was oxidized to produce beef tallow residue such as (E,E)-2,4-decadienal [25]. Moreover, the threshold of volatile aldehyde components was low. They contributed substantially to flavor due to their additive effect [26]. The significant decrease of (E,E)-2,4-decadienal in MRPs E might be due to its degradation by the reverse aldol condensation reactions with the prolongation of the hydrolysis time [27] and consumption by the Maillard reaction [28]. Benzene acetaldehyde produced a plant-like odor at low concentrations. However, high concentrations produce a waxy odor, decreasing sensory evaluation scores [29]. The lowest benzene acetaldehyde was present in MRPs C, contributing to the lowest rancidity. Some researchers suggested that unsaturated aldehydes during lipid oxidation cause phenylalanine degradation in response to hydrolysis time [30], resulting in increased benzene acetaldehyde content in MRPs D and E. This high benzene acetaldehyde content in our results might be due to beef’s high phenylalanine content in beef tallow residues. The (E)-2- octenal provides a fatty flavor primarily with a low FD value and a high content in MRPs C. Through oxidation, Oleic, linoleic and linolenic acids produce 9-hydroperoxide, which is capable of generating (E)-2-octenal via β-shearing [31,32].

There are three isomers of ethyl dimethyl pyrazine, among which 2-ethyl-3,5-dimethylpyrazine has been identified as an essential flavor component of roasted beef, roasted coffee, popcorn and roasted sesame [33]. Among the 30 volatile components, 2-ethyl-3,5-dimethylpyrazine had the highest FD values, and excessive or insufficient hydrolysis time resulted in a decrease in the 2- ethyl-3,5-dimethylpyrazine content, which was responsible for the most potent roast flavor found in MRPs C. Alanine was an essential precursor of fragrance-related trialkyl pyrazines such as 2-ethyl-3,5-dimethylpyrazine and 2,3-diethyl-5-methylpyrazine [33]. In addition, threonine and reducing sugar can also generate 2-ethyl-3,5-dimethylpyrazine and 2-ethyl-3,6-dimethylpyrazine through the Maillard reaction under specific temperature and pressure conditions [34]. In oxidative degradation of unsaturated fatty acids, alcohols are formed by positional isomerization of double bonds in the carbon chain [35]. The content of 1-octen-3-ol increased initially then decreased but then increased again due to prolonging hydrolysis time, similar to the findings of the research by other scholars [6]. The reason might be that 1-octene-3-ol (mushroom) is the typical alcohol produced during frying and decomposes in linoleic acid 10-hydroperoxide [36]. It was deduced that 1-octene-3-ol was produced during the frying process of suet.

Furan compounds represented a large class of heterocycles formed as intermediates or products in heat-induced reactions and could significantly affect the organoleptic properties of foods [37]. Of the five MRPs, 2-pentylfuran provided the fattiest and fruity flavors. A typical furan produced during frying is 2-pentyl-furan, formed when linoleic acid is oxidized by hydroperoxide [38]. The 2-pentylfuran was the most hydrolyzed after four hours, possibly due to hydroperoxide degradation. By the secondary decomposition of lipid oxidation products (such as hexanal and (E,E)-2,4-decadienal), carboxylic acids could be produced [39]. They generally had a bitter, rancid flavor that gave the MRPs a bad flavor, significantly affecting them when hydrolyzed for 2 h. These findings were consistent with some other research [40]. Carboxylic acid concentrations were consistent except for hexanoic acid, which had a fatty odor and was formed by the secondary degradation of hexanal and (E,E)-2,4-decadienal. Increasing hexanoic acid in MRPs B, C, D, and E may be related to the degradation of hexanal and (E,E)-2, 4-decadienal with prolonged hydrolysis time.

ROAVs were proposed to describe how volatile compounds contribute to the overall flavor based on their relative concentration, and the content of each volatile component could be calculated without internal standards. The result is more intuitive, and the error of internal standard is also eliminated. Therefore, ROVA is simpler and more convenient than odor activity values (OAVs) [41]. Additionally, the volatile components with ROAVs ≥ 1, such as 2-ethyl-3,5-dimethylpyrazine, played an essential role in the flavor of MRPs. Moreover, the volatile components with 0.1 ≤ ROAVs ≤ 1, such as hexanal, played an important role in flavor modification. However, the volatile components with ROAVs ≤ 0.1 such as (E)-2-undecenal played a weak role in the flavor of MRPs [42]. In the end, the overall flavor of the MRPs results from the interaction of the various volatile components. Due to the higher peak areas and relative content, (E,E)-3,5-octadien-2-one and ethyl myristate were significant compounds of MRPs. Despite their higher odor threshold, these aroma-active compounds showed low ROAVs. On the other hand, 2-ethyl-3,5-dimethylpyrazine and (E,E)-2,4-nonadienal had low relative amounts but had high ROAVs and strong flavor effects because of low thresholds. Therefore, the effect of volatile components on flavor was determined by combining content and threshold, consistent with Xu et al. [16].

This research exhibited that too short or too long hydrolysis times could increase the ROAVs of harmful components and worsen sensory evaluation. Results showed that the sensory evaluation score with DH of 11.93 had the best overall score. MRPs may produce different levels of volatile compounds and different intensities of individual sensory attributes with different DHs, in line with the results of Zhan et al. [43]. There is a possibility that different hydrolysis times produce different volatile components, which will affect Maillard reactions and result in different flavors of MRPs.

A rough estimate of ROAVs can be obtained by fitting proxy models to the key volatile components with an extension of hydrolysis time; the reason for not choosing 2-ethyl-3,5-dimethylpyrazine was that it was 100 in each group, so the curve simulation must also be a straight line. The CPM performed best because curve fitting required a large amount of data. The CPM could generate a large amount of prediction data from a small amount of experimental data, thus improving accuracy because PCF was based on uniformly distributing known points along two sides of a curve. Its accuracy was lower than CPM. It became impossible to fit a curve with high precision when the dispersion of the points was large. Some scholars used the PCF method to identify the key flavor compounds in dog food [44]. The fitting effect was better than that in this paper. In their study, the experimental data were monotonically increased. The data could fall on each side of the straight line equally. A PCF differs from the other two methods in that the CSI and CPM require the curve to pass through every given point. As opposed to the PCF, the resulting curve must be close to each given point but not necessarily pass through them, which was consistent with the results of Ge et al. [45]. The precision of CSI was the lowest due to fewer sample points available and a few functional relations to limit the function. Hence the boundary of the function could not be standardized, which would lead to the function of volatility [46]. CSI was also used to reconstruct the path concentration distribution of optical remote sensing measurement in a study carried out by Wu et al. [47]. This is probably because the data in that study can be seen as a simple model, while the problem in this study is much more complex.

There were significant differences in proxy models’ fitting degrees of key volatile components. Each prediction function was accurate for heptanal and (E)-2-octenal due to the increasing trend of all five points in the heptanal group. The concentration of data in the (E)-2-octenal group also resulted in accurate predictions. The poor prediction for (E)-2-nonanal and (E,E)-2,4-nonadienal might be due to the large numerical difference and fluctuation of the five points in the (E)-2-nonnal and (E,E)-2,4-nonadienal group, that is why the curve has many possible styles. At different time points, the proxy model predicted the states of the key volatile components. CPM was the most accurate, closest to the real values compared to the other two proxy models. This was ideal for modelling and simulating the Maillard reaction of the beef tallow residue hydrolysis product. The CPM fitting curve with the highest accuracy showed that the ROAVs of benzene acetaldehyde (waxy), (E)-2-nonenal (cucumber), (E,E)-2,4-decadienal (bedbug) and 1-octen-3-ol (moldy) was relatively low in the MRPs C. GC-O-MS analysis showed that the relative content of 2-ethyl-3,5-dimethylpyrazine was the highest. Moreover, other flavor substances (such as fatty, fruity flavor active components) did not have any defects, further supporting MRPs C flavor’s superiority after four hours of hydrolysis.

Even though this proof-of-concept study evaluated the CPM approach for one model MRPs and nine volatile components, it has been shown to apply to most small point fittings. Additional work will be needed to validate and extend the CPM approach to other small point fittings.

## 5. Conclusions

This study showed that the four-hour hydrolysis of beef tallow residue hydrolysate could improve the flavor through the subsequent Maillard reaction. Based on the GC-O-MS results, beef tallow residue’s flavor was mainly composed of fatty, rancid, stale, green, roasty and fruity, and nine volatile components contributed significantly to the flavor of beef tallow residue. According to the results of GC-O-MS, the ROAVs of main flavor substances such as 2-ethyl-3,5-dimethylpyrazine in the four-hour hydrolysis were higher than those of the other four groups. The sensory evaluation showed that the four-hour enzymatic hydrolysis had the highest roasty and the lowest stale flavor. In addition, compared with PCF and CSI, the CPM has high predictability for the volatile ROAVs-time curve. Therefore, preparing MRPs C could improve the flavor, and the CPM could simulate the change curve of the volatile ROAVs-time of beef tallow residue.

## Figures and Tables

**Table 1:** Order contribution of volatile compounds of MRPs.

No.	Aroma-Active Compounds	Flavor Dilution Factor ^a^					Flavor ^b^
		MRPs A	MRPs B	MRPs C	MRPs D	MRPs E	
1	(E)-2-Octenal	9	9	27	9	9	fatty, plastic [16]
2	Octanal	3	9	9	9	27	fatty [16]
3	(E)-2-Decenal	1	3	9	3	3	fatty, green, waxy [16]
4	(E,E)-2,4-Nonadienal	9	9	9	9	3	fatty, green, waxy [16]
5	(E)-2-Undecenal	1	3	3	3	3	fatty, green, waxy [16]
6	2-Pentyl- furan	9	3	9	3	3	fatty, fruity, green [16]
7	1-Octanol	1	3	3	3	3	fatty, floral, green [16]
8	Decanal	3	3	3	9	9	fatty, floral, green [16]
9	(E)-2-Octen-1-ol	3	9	27	9	3	Sour, fatty, gravy [20]
10	3-(Methylthio)propanal	3	9	3	9	3	boiled potato [16]
11	n-Decanoic acid	3	3	3	3	3	rubber, sour [21]
12	(E,E)-2,4-Decadienal	9	27	9	9	9	bedbug [16]
13	Butanoic acid	ND ^c^	ND	1	1	1	rancid, moldy [16]
14	Octanoic acid	9	3	9	9	9	rancid, fermented [16]
15	(E,E)-3,5-Octadien-2-one	3	9	9	9	9	rancid [20]
16	Phenol	9	9	9	9	9	plastic, rancid [20]
17	1-Octen-3-ol	3	9	9	3	3	moldy [21]
18	Trimethyl-pyrazine	ND	ND	9	9	9	moldy, plastic [22]
19	Nonanoic acid	9	3	9	9	9	moldy, rancid [16]
20	2-Ethyl-6-methyl- pyrazine	1	1	3	3	3	peanut, roasted [16]
21	Ethyl myristate	9	3	3	3	3	peanut, rubber [20]
22	Hexanoic acid	1	1	1	1	1	rancid, bitter [16]
23	2-Ethyl-3,5-dimethylpyrazine	81	81	243	243	243	peanut, roasted [16]
24	Hexanal	1	1	1	1	1	green [16]
25	p-Cresol	ND	ND	1	9	3	herbal medicine [23]
26	(E)-2-Nonenal	27	27	27	27	27	fatty, mushroom [16]
27	Heptanal	9	9	27	27	27	citrus-like [16]
28	Heptanoic acid	1	1	1	1	1	sweaty [16]
29	Benzene acetaldehyde	27	27	27	27	27	stale, floral [16]
30	Nonanal	9	9	27	9	27	citrus-like, fatty [16]

^a^ FD factor was determined by AEDA on a DB-WAX capillary column; ^b^ the flavor was detected by GC-O with the reference [16,20,21,22,23]; ^c^ ND: not detected.

**Table 2:** The relative concentration of aroma-active compounds in MRPs.

No.	Aroma-Active Compounds	Relative Content (%) *					Ions (*m*/*z*)
		MRPs A	MRPs B	MRPs C	MRPs D	MRPs E	
1	(E)-2-Octenal	2.16 ^d^ ± 0.42	4.47 ^b^ ± 0.24	6.38 ^a^ ± 0.05	3.45 ^c^ ± 0.15	4.64 ^b^ ± 0.62	41, 55, 70
2	Octanal	0.82 ^d^ ± 0.02	1.37 ^c^ ± 0.28	1.76 ^b^ ± 0.07	1.74 ^b^ ± 0.33	2.35 ^a^ ± 0.20	41, 57, 84
3	(E)-2-Decenal	0.72 ^c^ ± 0.04	1.58 ^a^ ± 0.03	1.62 ^a^ ± 0.19	1.49 ^b^ ± 0.05	1.48 ^b^ ± 0.02	41, 70, 55
4	(E,E)-2,4-Nonadienal	0.38 ^c^ ± 0.03	1.17 ^a^ ± 0.12	0.51 ^b^ ± 0.02	0.31 ^c^ ± 0.04	0.12 ^d^ ± 0.01	81, 41, 67
5	(E)-2-Undecenal	0.74 ^b^ ± 0.02	1.47 ^a^ ± 0.16	1.29 ^a^ ± 0.05	1.49 ^a^ ± 0.03	1.37 ^a^ ± 0.05	41, 70, 55
6	2-Pentyl- furan	2.73 ^b^ ± 0.29	1.46 ^d^ ± 0.15	3.31 ^a^ ± 0.04	0.76 ^e^ ± 0.08	1.84 ^c^ ± 0.11	81, 53, 82
7	1-Octanol	0.42 ^d^ ± 0.11	0.62 ^c^ ± 0.04	0.72 ^b^ ± 0.05	0.93 ^a^ ± 0.12	0.90 ^a^ ± 0.08	56, 41, 69
8	Decanal	0.53 ^c^ ± 0.07	0.61 ^c^ ± 0.02	0.36 ^d^ ± 0.01	0.79 ^b^ ± 0.08	0.91 ^a^ ± 0.04	41, 43, 57
9	(E)-2-Octen-1-ol	0.75 ^c^ ± 0.17	1.10 ^b^ ± 0.05	2.55 ^a^ ± 0.02	1.19 ^b^ ± 0.11	0.84 ^c^ ± 0.03	41, 55, 83
10	3-(Methylthio) propanal	0.24 ^e^ ± 0.02	0.76 ^c^ ± 0.03	0.34 ^d^ ± 0.03	0.84 ^b^ ± 0.05	1.25 ^a^ ± 0.09	48, 104, 76
11	n-Decanoic acid	0.44 ^b^ ± 0.01	0.50 ^b^ ± 0.06	0.30 ^c^ ± 0.01	0.59 ^a^ ± 0.03	0.64 ^a^ ± 0.03	60, 73, 55
12	(E,E)-2,4-Decadienal	2.25 ^c^ ± 0.30	5.23 ^a^ ± 0.98	2.95 ^b^ ± 0.01	2.44 ^c^ ± 0.28	1.25 ^d^ ± 0.05	81, 41, 67
13	Butanoic acid	0.00 ^c^ ± 0.00	0.00 ^c^ ± 0.00	0.08 ^b^ ± 0.00	0.80 ^a^ ± 0.13	0.05 ^c^ ± 0.01	60, 73, 39
14	Octanoic acid	0.23 ^b^ ± 0.03	0.15 ^c^ ± 0.01	0.37 ^a^ ± 0.02	0.40 ^a^ ± 0.04	0.40 ^a^ ± 0.10	60, 73, 43
15	(E,E)-3,5-Octadien-2-one	0.38 ^d^ ± 0.07	0.53 ^c^ ± 0.05	1.84 ^a^ ± 0.13	0.74 ^b^ ± 0.05	0.84 ^b^ ± 0.19	55, 43, 125
16	Phenol	0.24 ^a^ ± 0.08	0.18 ^a^ ± 0.03	0.11 ^b^ ± 0.01	0.16 ^a^ ± 0.01	0.17 ^a^ ± 0.02	68, 40, 55
17	1-Octen-3-ol	1.94 ^b^ ± 0.53	4.34 ^a^ ± 0.65	3.94 ^a^ ± 0.09	1.69 ^c^ ± 0.35	2.48 ^b^ ± 0.20	56, 41, 59
18	Trimethyl-pyrazine	0.00 ^c^ ± 0.00	0.00 ^c^ ± 0.00	0.48 ^b^ ± 0.03	0.48 ^b^ ± 0.07	0.72 ^a^ ± 0.07	121, 67, 80
19	Nonanoic acid	0.32 ^b^ ± 0.03	0.17 ^d^ ± 0.01	0.40 ^b^ ± 0.07	0.40 ^b^ ± 0.03	0.52 ^a^ ± 0.03	60, 73, 41
20	2-Ethyl-6-methyl- pyrazine	0.16 ^c^ ± 0.01	0.16 ^c^ ± 0.02	0.94 ^b^ ± 0.09	1.00 ^b^ ± 0.12	1.28 ^a^ ± 0.20	42, 108, 39
21	Ethyl myristate	2.20 ^a^ ± 0.36	1.30 ^b^ ± 0.33	0.98 ^b^ ± 0.02	1.19 ^b^ ± 0.15	1.13 ^b^ ± 0.13	77, 106, 51
22	Hexanoic acid	0.46 ^e^ ± 0.17	2.56 ^b^ ± 0.05	2.24 ^c^ ± 0.07	2.89 ^a^ ± 0.17	1.56 ^d^ ± 0.17	60, 73, 41
23	2-Ethyl-3,5-dimethylpyrazine	0.59 ^c^ ± 0.03	0.50 ^d^ ± 0.03	1.12 ^a^ ± 0.03	0.87 ^b^ ± 0.32	0.85 ^b^ ± 0.07	135, 56, 39
24	Hexanal	0.45 ^a^ ± 0.09	0.36 ^b^ ± 0.01	0.38 ^b^ ± 0.02	0.31 ^c^ ± 0.01	0.51 ^a^ ± 0.03	44, 56, 41
25	p-Cresol	0.00 ^c^ ± 0.00	0.00 ^c^ ± 0.00	0.00 ^c^ ± 0.00	0.67 ^a^ ± 0.03	0.28 ^b^ ± 0.02	43, 57, 128
26	(E)-2-Nonenal	0.68 ^d^ ± 0.10	1.66 ^b^ ± 0.22	1.31 ^b^ ± 0.15	2.97 ^a^ ± 0.32	1.19 ^c^ ± 0.03	41, 55, 70
27	Heptanal	0.57 ^c^ ± 0.08	0.57 ^c^ ± 0.07	1.38 ^b^ ± 0.04	1.44 ^b^ ± 0.45	1.84 ^a^ ± 0.17	70, 55, 44
28	Heptanoic acid	0.06 ^b^ ± 0.00	0.06 ^b^ ± 0.00	0.07 ^b^ ± 0.02	0.07 ^b^ ± 0.02	0.11 ^a^ ± 0.01	60, 73, 87
29	Benzene acetaldehyde	5.57 ^a^ ± 0.87	2.97 ^b^ ± 0.83	1.83 ^c^ ± 0.07	5.62 ^a^ ± 1.33	4.09 ^a^ ± 0.87	91, 120, 65
30	Nonanal	4.95 ^c^ ± 0.93	4.65 ^c^ ± 0.75	8.37 ^a^ ± 0.24	4.49 ^c^ ± 0.21	6.90 ^b^ ± 0.69	41, 57, 70

* Values bearing different lowercase letters (a, b, c, d and e) were significantly different (*p* ≤ 0.5).

**Table 3:** Relative odor activity values of aroma-active compounds in MRPs.

No.	Aroma-Active Compounds	RI ^a^	Odor Threshold in Water (µg/kg) ^b^	ROAV				
				MRPs A	MRPs B	MRPs C	MRPs D	MRPs E
1	(E,E)-3,5-Octadien-2-one	1068	0.1	0.007	0.010	0.016	0.009	0.010
2	Hexanal	1087	0.0011	0.687	0.644	0.309	0.32	0.548
3	Heptanal	1182	0.0009	1.066	1.252	1.369	1.848	2.394
4	2-Pentyl- furan	1235	0.019	0.244	0.153	0.156	0.046	0.113
5	p-Cresol	1251	0.0084	0	0	0	0.092	0.039
6	Octanal	1291	0.0004	3.48	6.801	3.929	5.023	6.876
7	Ethyl myristate	1322	0.18	0.021	0.014	0.005	0.008	0.007
8	2-Ethyl-3,5-dimethylpyrazine	1346	0.00001	100	100	100	100	100
9	2-Ethyl-6-methyl-pyrazine	1371	0.04	0.007	0.008	0.021	0.029	0.037
10	Nonanal	1395	0.0026	3.234	3.545	2.874	1.992	3.113
11	Trimethyl-pyrazine	1402	0.033	0	0	0.013	0.017	0.026
12	(E)-2-Octenal	1426	0.0027	1.359	3.286	2.11	1.475	2.013
13	1-Octen-3-ol	1438	0.0027	1.222	3.191	1.303	0.723	1.076
14	3-(Methylthio)propanal	1454	0.0014	0.298	1.083	0.214	0.69	1.047
15	Decanal	1497	0.0026	0.352	0.466	0.124	0.35	0.409
16	(E)-2-Nonenal	1531	0.00009	12.884	36.53	12.996	38.106	15.54
17	(E)-2-Octen-1-ol	1544	0.04	0.032	0.055	0.057	0.034	0.025
18	1-Octanol	1559	0.022	0.032	0.056	0.029	0.049	0.048
19	Butanoic acid	1631	0.004	0	0	0.017	0.23	0.015
20	Benzene acetaldehyde	1643	0.0017	5.564	3.462	0.96	3.818	2.818
21	(E)-2-Decenal	1654	0.0027	0.457	1.162	0.536	0.636	0.644
22	(E,E)-2,4-Nonadienal	1778	0.0002	3.285	11.567	2.277	1.807	0.686
23	(E)-2-Undecenal	1861	0.044	0.029	0.066	0.026	0.039	0.036
24	(E,E)-2,4-Decadienal	2001	0.0023	1.665	4.514	1.145	1.227	0.638
25	Phenol	2020	0.046	0.009	0.008	0.002	0.004	0.004
26	Hexanoic acid	2050	0.04	0.02	0.127	0.05	0.083	0.046
27	Heptanoic acid	2130	0.022	0.005	0.006	0.003	0.004	0.006
28	Octanoic acid	2264	0.0051	0.077	0.058	0.065	0.091	0.092
29	n-Decanoic acid	2276	0.05	0.015	0.02	0.005	0.014	0.015
30	Nonanoic acid	2370	0.02	0.027	0.017	0.018	0.023	0.031

^a^ RIs were determined using a homologous series of n-alkanes on DB-WAX capillary columns; ^b^ odor thresholds were from the reference [24].

**Table 4:** Prediction error of three proxy models in verification experiment.

Aroma-Active Compounds	Relative Content				Error		
	Actual Measured	PCF	CSI	CPM	PCF	CSI	CPM
(E)-2-Octenal	1.682	2.263	1.895	1.638	0.581	0.213	0.044
Octanal	3.972	5.361	3.971	3.972	1.389	0.001	0
(E,E)-2,4-Nonadienal	1.032	4.265	−21.173	0.971	3.233	22.205	0.061
(E,E)-2,4-Decadienal	0.843	1.966	−6.910	0.803	1.123	7.753	0.040
1-Octen-3-ol	0.826	1.568	−3.742	0.791	0.742	4.568	0.035
(E)-2-Nonenal	20.535	28.317	−45.821	22.492	7.782	66.356	1.957
Heptanal	1.576	1.646	1.394	1.575	0.070	0.182	0.001
Benzene acetaldehyde	1.994	2.254	0.042	2.061	0.260	1.952	0.067
Nonanal	2.295	2.722	1.421	2.344	0.427	0.874	0.049

## Data Availability

Data is contained within the article.
